# Supplementary material for: Artificial intelligence-aided protein engineering: from topological data analysis to deep protein language models
Source: ArXiv. 2023 Jul 27:arXiv:2307.14587v1. Preprint. [Version 1] (PMC10402185)
Supplement: 1 [file NIHPP2307.14587V1-supplement-1.pdf]

# Mathematical theory of topological data analysis (TDA)

## Simplicial complex and chain complex

Graph is a representation for a point cloud consisting of vertices and edges for modeling pairwise interactions, such as atoms and bonds in molecules. Simplicial complex, the generalization of graph, constructs more enriched shapes to include high dimensional objects. A simplicial complex is composed of simplexes up to certain dimensions. A $k$-simplex, $\sigma^k$, is a convex hull of $k+1$ affinely independent points $v_0, v_1, v_2, \cdots, v_k$:

$$\sigma^k := [v_0, v_1, v_2, \cdots, v_k] = \left\{ \sum_{i=0}^{k} \lambda_i v_i \middle| \sum_{i=0}^{k} \lambda_i = 1; \lambda_i \in [0,1], \ \forall i \right\}. \tag{4}$$

In Euclidean space, 0-simplex is a point, 1-simplex is an edge, 2-simplex is a triangle, and 3-simplex is a tetrahedron. The $k$-simplex can describe abstract simplex for $k > 3$.

A subset of the $k+1$ vertices of a $k$-simplex, $\sigma^k$, with $m+1$ vertices forming a convex hull in a lower dimension and is called an $m$-face of the $k$-simplex $\sigma^m$, denoted as $\sigma^m \subset \sigma^k$. A simplicial complex $K$ is a finite collection of simplexes satisfying two conditions:

1) Any face of a simplex in $K$ is also in $K$.

2) The intersection of any two simplexes in $K$ is either empty or a shared face.

The interactions between two simplexes can be described by adjacency. For example, in graph theory, two vertices (0-simplexes) are adjacent if they share a common edge (1-simplex). Adjacency for $k$-simplexes with $k > 0$ includes both upper and lower adjacency. Two distinct $k$-simplexes, $\sigma_1$ and $\sigma_2$, in $K$ are upper adjacent, denoted $\sigma_1 \sim_U \sigma_2$, if both are faces of a $(k+1)$-simplex in $K$, called a common upper simplex. Two distinct $k$-simplexes, $\sigma_1$ and $\sigma_2$, in $K$ are lower adjacent, denoted $\sigma_1 \sim_L \sigma_2$, if they share a common $(k-1)$-simplex as their face, called a common lower simplex. Either common upper simplex or common lower simplex is unique for two upper or lower adjacent simplexes. The upper degree of a $k$-simplex, $\deg_U(\sigma^k)$, is the number of $(k+1)$-simplexes in $K$ of which $\sigma^k$ is a face; the lower degree of a $k$-simplex, $\deg_L(\sigma^k)$, is the number of nonempty $(k-1)$-simplexes in $K$ that are faces of $\sigma^k$, which is always $k+1$. The degree of $k$-simplex ($k > 0$) is defined as the sum of its upper and lower degree

$$\deg(\sigma^k) = \deg_U(\sigma^k) + \deg_L(\sigma^k) = \deg_U(\sigma^k) + k + 1. \tag{5}$$

For $k = 0$, the degree of a vertex is:

$$\deg(\sigma^0) = \deg_U(\sigma^0). \tag{6}$$

A simplex has orientation determined by the ordering of its vertices, except 0-simplex. For example, clockwise and anticlockwise orderings of three vertices determine the two orientation of a triangle. Two simplexes, $\sigma_1$ and $\sigma_2$, defined on the same vertices are similarly oriented if their orderings of vertices differ from an even number of permutations, otherwise, they are dissimilarly oriented.

Algebraic topology provides a tool to calculate simplicial complex. A $k$-chain is a formal sum of oriented $k$-simplexes in $K$ with coefficients on $\mathbb{Z}$. The set of all $k$-chains of simplicial complex $K$ together with the addition operation on $\mathbb{Z}$ constructs a free Abelian group $C_k(K)$, called chain group. To link chain groups from different dimensions, the $k$-boundary operator, $\partial_k : C_k(K) \to C_{k-1}(K)$, maps a $k$-chain in the form of a linear combination of $k$-simplexes to the same linear combination of the boundaries of the $k$-simplexes. For a simple example where the $k$-chain has one oriented $k$-simplex spanned by $k+1$ vertices as defined in Eq. (4), its boundary operator is defined as the formal sum of its all $(k-1)$-faces:

$$\partial_k \sigma^k = \sum_{i=0}^{k} (-1)^i \sigma_i^{k-1} = \sum_{i=0}^{k} (-1)^i [v_0, \cdots, \hat{v_i}, \cdots, v_k], \tag{7}$$

where $\sigma_i^{k-1} = [v_0, \cdots, \hat{v_i}, \cdots, v_k]$ is the $(k-1)$-simplex with its vertex $v_i$ being removed. The most important topological property is that a boundary has no boundary: $\partial_{k-1}\partial_k = \emptyset$.

A sequence of chain groups connected by boundary operators defines the chain complex:

$$\cdots \xrightarrow{\partial_{n+1}} C_n(K) \xrightarrow{\partial_n} C_{n-1}(K) \xrightarrow{\partial_{n-1}} \cdots \xrightarrow{\partial_1} C_0(K) \xrightarrow{\partial_0} \emptyset. \tag{8}$$

When $n$ exceeds the dimension of $K$, $C_n(K)$ is an empty vector space and the corresponding boundary operator is a zero map.

## Filtration for multiscale chain complexes

Filtration is a process that constructs a nested sequence of simplicial complex allowing a multiscale analysis of the point cloud. It creates a family of simplicial complexes ordered by inclusion (Figure 2c):

$$\emptyset = K^{t_0} \subseteq K^{t_1} \subseteq \cdots \subseteq K^{t_n} = K. \tag{9}$$

where $K$ is the largest simplicial complex can be obtained from the point cloud.

The filtration induces a sequence of chain complexes

$$\begin{array}{ccccccccc}
\cdots \underset{\partial_{k+2}^{t_1 *}}{\overset{\partial_{k+2}^{t_1}}{\rightleftarrows}} & C_{k+1}^{t_1} & \underset{\partial_{k+1}^{t_1 *}}{\overset{\partial_{k+1}^{t_1}}{\rightleftarrows}} & C_k^{t_1} & \underset{\partial_{k}^{t_1 *}}{\overset{\partial_{k}^{t_1}}{\rightleftarrows}} \cdots \underset{\partial_{1}^{t_1 *}}{\overset{\partial_{1}^{t_1}}{\rightleftarrows}} & C_0^{t_1} & \underset{\partial_{0}^{t_1 *}}{\overset{\partial_{0}^{t_1}}{\rightleftarrows}} \emptyset \\
& \cap\!| & & \cap\!| & & \cap\!| & \\
\cdots \underset{\partial_{k+2}^{t_2 *}}{\overset{\partial_{k+2}^{t_2}}{\rightleftarrows}} & C_{k+1}^{t_2} & \underset{\partial_{k+1}^{t_2 *}}{\overset{\partial_{k+1}^{t_2}}{\rightleftarrows}} & C_k^{t_2} & \underset{\partial_{k}^{t_2 *}}{\overset{\partial_{k}^{t_2}}{\rightleftarrows}} \cdots \underset{\partial_{1}^{t_2 *}}{\overset{\partial_{1}^{t_2}}{\rightleftarrows}} & C_0^{t_2} & \underset{\partial_{0}^{t_2 *}}{\overset{\partial_{0}^{t_2}}{\rightleftarrows}} \emptyset \\
& \cap\!| & & \cap\!| & & \cap\!| & \\
& \vdots & & \vdots & & \vdots & \\
& \cap\!| & & \cap\!| & & \cap\!| & \\
\cdots \underset{\partial_{k+2}^{t_n *}}{\overset{\partial_{k+2}^{t_n}}{\rightleftarrows}} & C_{k+1}^{t_n} & \underset{\partial_{k+1}^{t_n *}}{\overset{\partial_{k+1}^{t_n}}{\rightleftarrows}} & C_k^{t_n} & \underset{\partial_{k}^{t_n *}}{\overset{\partial_{k}^{t_n}}{\rightleftarrows}} \cdots \underset{\partial_{1}^{t_n *}}{\overset{\partial_{1}^{t_n}}{\rightleftarrows}} & C_0^{t_n} & \underset{\partial_{0}^{t_n *}}{\overset{\partial_{0}^{t_n}}{\rightleftarrows}} \emptyset
\end{array} \tag{10}$$

where $C_k^t = C_k(K^t)$ is the chain group for subcomplex $K^t$, and its $k$-boundary operator is $\partial_k^t : C_k(K^t) \to C_{k-1}(K^t)$. $\partial_k^t$ is the co-boundary operator. Associated with the $k$-boundary operator, its adjoint operator is the $k$-adjoint boundary operator, $\partial_k^{t*} : C_{k-1}(K^t) \to C_k(K^t)$.

There are various simplicial complex that can be used to construct the filtration, such as Rips complex, Čech complex, and Alpha complex. For example, the Rips complex of $K$ with radius $t$ consists of all simplexes with diameter at most $2t$:

$$V(t) = \{\sigma \subseteq K | \text{diam}(\sigma) \leq 2t\}. \tag{11}$$

## Homology group and persistent homology

With the chain complex defined in Eq. (8), the $k$-cycle and $k$-boundary groups are defined as:

$$\begin{aligned} Z_k &= \ker\, \partial_k = \{c \in C_k \mid \partial_k c = 0\} \\ B_k &= \mathrm{im}\, \partial_{k+1} = \{\partial_{k+1} c \mid c \in C_{k+1}\} \end{aligned} \tag{12}$$

Then the $k$-th homology group $H_k$ is defined as

$$H_k = Z_k / B_k. \tag{13}$$

The $k$-th Betti number, $\beta_k$, is defined by the rank of $k$-th homology group $H_k$ which counts $k$-dimensional holes. For example, $\beta_0 = \mathrm{rank}(H_0)$ reflects the number of connected components, $\beta_1 = \mathrm{rank}(H_1)$ reflects the number of loops, and $\beta_2 = \mathrm{rank}(H_2)$ reveals the number of voids or cavities.

Persistent homology is devised to track the multiscale topological information along the filtration [1]. The inclusion map $K_i \subseteq K_j$ induces a homomorphism $f_k^{i,j}$ between homology groups $H_k(K_{t_i}) \rightarrow H_k(K_{t_j})$ for each dimension $k$. The $p$-persistent $k$-th homology group of $K_t$ is defined by

$$H_k^{t,p} = Z_k^t / (B_k^{t+p} \cap Z_k^t), \tag{14}$$

where $Z_k^t = \ker\, \partial_k^t$ and $B_k^{t+p} = \mathrm{im}\, \partial_{k+1}^{t+p}$. Intuitively, this homology group records the $k$-dimensional homology classes of $K_t$ that are persistent at least until $K_{t+p}$. The birth and death of homology classes can be represented by a barcode, a set of intervals (Figure 2d).

## Combinatorial Laplacian.

For $k$-boundary operator $\partial_k : C_k \rightarrow C_{k-1}$ in $K$, let $\mathcal{B}_k$ be the matrix representation of this operator relative to the standard bases for $C_k$ and $C_{k-1}$ in $K$. $\mathcal{B}_k \in \mathbb{Z}^{M\times N}$ is the matrix representation of boundary operator under the standard bases $\left\{\sigma_i^k\right\}_{i=1}^N$ and $\left\{\sigma_j^{k-1}\right\}_{j=1}^M$ of $C_k$ and $C_{k-1}$. Associated with the boundary operator $\partial_k$, the adjoint boundary operator is $\partial_k^* : C_{k-1} \rightarrow C_k$, where its matrix representation is the transpose of the matrix, $\mathcal{B}^T$, with respect to the same ordered bases to the boundary operator.

The $k$-combinatorial Laplacian, a topological Laplacian, is a linear operator $\Delta_k : C_k(K) \rightarrow C_k(K)$

$$\Delta_k := \partial_{k+1}\partial_{k+1}^* + \partial_k^*\partial_k, \tag{15}$$

and its matrix representation, $L_k$, is given by

$$L_k = \mathcal{B}_{k+1}\mathcal{B}_{k+1}^T + \mathcal{B}_k^T\mathcal{B}_k. \tag{16}$$

In particular, the 0-combinatorial Laplacian (i.e. graph Laplacian) is given as follows since $\partial_0$ is an zero map:

$$L_0 = \mathcal{B}_1\mathcal{B}_1^T. \tag{17}$$

The elements of $k$-combinatorial Laplaicn matrices are

$$(L_k)_{i,j} = \begin{cases} \deg\left(\sigma_i^k\right), & \text{if } i = j \\ 1, & \text{if } i \neq j, \sigma_i^k \nsim_U \sigma_j^k \text{ and } \sigma_i^k \sim_L \sigma_j^k \text{ with similar orientation} \\ -1, & \text{if } i \neq j, \sigma_i^k \nsim_U \sigma_j^k \text{ and } \sigma_i^k \sim_L \sigma_j^k \text{ with dissimilar orientation} \\ 0, & \text{if } i \neq j, \text{ either } \sigma_i^k \sim_U \sigma_j^k \text{ or } \sigma_i^k \nsim_L \sigma_j^k. \end{cases} \tag{18}$$

For $k = 0$, the graph Laplacian matrix $L_0$ is

$$(L_0)_{i,j} = \begin{cases} \deg\left(\sigma_i^0\right), & \text{if } i = j \\ -1, & \text{if } i \neq j, \sigma_i^0 \sim_U \sigma_j^0 \\ 0, & \text{otherwise.} \end{cases} \tag{19}$$

The multiplicity of zero spectra of $L_k$ gives the Betti-$k$ number, according to combinatorial Hodge theorem [2]:

$$\beta_k = \dim(L_k) - \mathrm{rank}(L_k) = \mathrm{null}(L_k). \tag{20}$$

The Betti numbers describe topological invariants. Specifically, $\beta_0$, $\beta_1$, and $\beta_2$ may be regarded as the numbers of independent components, rings, and cavities, respectively.

## Persistent spectral graph (PSG)

The homotopic shape changes with a small increment of filtration parameter may be subject to noise from the data. The persistence may be considered to enhance the robustness when calculating the Laplacian. First, we define the $p$-persistent chain group $\mathbb{C}_k^{t,p} \subseteq C_k^{t+p}$ whose boundary is in $C_{k-1}^t$:

$$\mathbb{C}_k^{t,p} = \left\{\alpha \in C_k^{t+p} \mid \partial_k^{t+p}(\alpha) \in C_{k-1}^t\right\}, \tag{21}$$

where $\partial_k^{t+p} : C_k^{t+p} \rightarrow C_{k-1}^{t+p}$ is the $k$-boundary operator for chain group $C_k^{t+p}$. Then we can define a $p$-persistent boundary operator, $\eth_k^{t,p}$, as the restriction of $\partial_k^{t+p}$ on the $p$-persistent chain group $\mathbb{C}_k^{t,p}$:

$$\eth_k^{t,p} = \partial_k^{t+p}|_{\mathbb{C}_k^{t,p}} : \mathbb{C}_k^{t,p} \rightarrow C_{k-1}^t.S \tag{22}$$

Then PSG defines a family of $p$-persistent $k$-combinatorial Laplacian operators $\Delta_k^{t,p} : C_k(K_t) \rightarrow C_k(K_t)$ [3, 4] which is defined as

$$\Delta_k^{t,p} = \eth_{k+1}^{t,p}\left(\eth_{k+1}^{t,p}\right)^* + \left(\partial_k^t\right)^*\partial_k^t. \tag{23}$$

We denote $\mathcal{B}_{k+1}^{t,p}$ and $\mathcal{B}_k^t$ as the matrix representations for boundary operators $\eth_{k+1}^{t,p}$ and $\partial_k^t$, respectively. Then the Laplacian matrix for $\Delta_k^{t,p}$ is

$$\mathcal{L}_k^{t,p} = \mathcal{B}_{k+1}^{t,p}\left(\mathcal{B}_{k+1}^{t,p}\right)^T + \left(\mathcal{B}_k^t\right)^T\mathcal{B}_k^t. \tag{24}$$

Since the Laplacian matrix, $\mathcal{L}_k^{t,p}$, is positive-semidefinite, its spectra are all real and non-negative

$$S_k^{t,p} = \mathrm{Spectra}(\mathcal{L}_k^{t,p}) = \{(\lambda_1)_k^{t,p}, (\lambda_2)_k^{t,p}, \cdots, (\lambda_N)_k^{t,p}\}, \tag{25}$$

where $N$ is the dimension of a standard basis for $C_k^t$, and $\mathcal{L}_k^{t,p}$ has dimension $N \times N$. The $k$-persistent Betti number $\beta_k^{t,p}$ can be obtained from the multiplicity of harmonic spectra of $\mathcal{L}_k^{t,p}$:

$$\beta_k^{t,p} = \dim(\mathcal{L}_k^{t,p}) - \mathrm{rank}(\mathcal{L}_k^{t,p}) = \mathrm{null}(\mathcal{L}_k^{t,p}) = \#\{i | (\lambda_i)_k^{t,p} \in S_k^{t,p}, \text{ and } (\lambda_i)_k^{t,p} \text{ [illegible]} \tag{26}$$

In addition, the rest of the spectra, i.e., the non-harmonic part, capture additional geometric information. The family of spectra of the persistent Laplacians reveals the homotopic shape evolution [5].

## References

1. Herbert Edelsbrunner, David Letscher, and Afra Zomorodian. Topological persistence and simplification. In *Proceedings 41st annual symposium on foundations of computer science*, pages 454–463. IEEE, 2000.
2. Beno Eckmann. Harmonische funktionen und randwertaufgaben in einem komplex. *Commentarii Mathematici Helvetici*, 17(1):240–255, 1944.
3. Rui Wang, Duc Duy Nguyen, and Guo-Wei Wei. Persistent spectral graph. *International journal for numerical methods in biomedical engineering*, 36(9):e3376, 2020.
4. Facundo Mémoli, Zhengchao Wan, and Yusu Wang. Persistent laplacians: Properties, algorithms and implications. *SIAM Journal on Mathematics of Data Science*, 4(2):858–884, 2022.
5. Yuchi Qiu and Guo-Wei Wei. Persistent spectral theory-guided protein engineering. *Nature Computational Science*, pages 1–15, 2023.
